# Validating a Machine Learning Algorithm to Predict 30-Day Re-Admissions in Patients With Heart Failure: Protocol for a Prospective Cohort Study

**DOI:** 10.2196/resprot.9466

**Published:** 2018-09-04

**Authors:** Sujay Kakarmath, Sara Golas, Jennifer Felsted, Joseph Kvedar, Kamal Jethwani, Stephen Agboola

**Affiliations:** ^1^ Data Science and Analytics Unit Partners Connected Health Partners HealthCare Boston, MA United States; ^2^ Department of Dermatology Massachusetts General Hospital Boston, MA United States; ^3^ Department of Dermatology Harvard Medical School Boston, MA United States; ^4^ Partners Connected Health Partners HealthCare Boston, MA United States

**Keywords:** electronic medical records, heart failure, machine learning, predictive algorithms, readmissions

## Abstract

**Background:**

Big data solutions, particularly machine learning predictive algorithms, have demonstrated the ability to unlock value from data in real time in many settings outside of health care. Rapid growth in electronic medical record adoption and the shift from a volume-based to a value-based reimbursement structure in the US health care system has spurred investments in machine learning solutions. Machine learning methods can be used to build flexible, customized, and automated predictive models to optimize resource allocation and improve the efficiency and quality of health care. However, these models are prone to the problems of overfitting, confounding, and decay in predictive performance over time. It is, therefore, necessary to evaluate machine learning–based predictive models in an independent dataset before they can be adopted in the clinical practice. In this paper, we describe the protocol for independent, prospective validation of a machine learning–based model trained to predict the risk of 30-day re-admission in patients with heart failure.

**Objective:**

This study aims to prospectively validate a machine learning–based predictive model for inpatient admissions in patients with heart failure by comparing its predictions of risk for 30-day re-admissions against outcomes observed prospectively in an independent patient cohort.

**Methods:**

All adult patients with heart failure who are discharged alive from an inpatient admission will be prospectively monitored for 30-day re-admissions through reports generated by the electronic medical record system. Of these, patients who are part of the training dataset will be excluded to avoid information leakage to the algorithm. An expected sample size of 1228 index admissions will be required to observe a minimum of 100 30-day re-admission events. Deidentified structured and unstructured data will be fed to the algorithm, and its prediction will be recorded. The overall model performance will be assessed using the concordance statistic. Furthermore, multiple discrimination thresholds for screening high-risk patients will be evaluated according to the sensitivity, specificity, predictive values, and estimated cost savings to our health care system.

**Results:**

The project received funding in April 2017 and data collection began in June 2017. Enrollment was completed in July 2017. Data analysis is currently underway, and the first results are expected to be submitted for publication in October 2018.

**Conclusions:**

To the best of our knowledge, this is one of the first studies to prospectively evaluate a predictive machine learning algorithm in a real-world setting. Findings from this study will help to measure the robustness of predictions made by machine learning algorithms and set a realistic benchmark for expectations of gains that can be made through its application to health care.

**Registered Report Identifier:**

RR1-10.2196/9466

## Introduction

Big data solutions, particularly machine learning predictive algorithms, have demonstrated the ability to unlock value from large, complex data in real time in aviation, astronomy, transportation, education, marketing, news, finance, publishing, and even entertainment. The health care industry, on the other hand, is often widely regarded as a late adopter of big data solutions. In the United States, at least one of the reasons that contributed to this delay is the relatively low adoption of electronic medical records (EMRs) among hospitals. In 2008, the number of hospitals that had a basic EMR system was 9%; by 2015, this number grew to 96% [1]. The rapid growth in the EMR adoption, coupled with the shift in the US health care system from a volume-based to a value-based reimbursement structure has spurred investments into artificial intelligence (AI)–based solutions for health care problems [2].

The hospital re-admission rate is one of the metrics used to measure the quality of care provided by a hospital [3,4]. In the financial year 2017, the Centers for Medicare and Medicaid Services withheld more than US $500 million in payments to 2597 hospitals in the United States under its re-admissions reduction program [5]. Naturally, hospitals have begun implementing various interventions to reduce re-admission rates [6]. To optimize the use of expensive care transition interventions, one of the strategies adopted by hospitals has been to focus on patients predicted to be at a higher risk of re-admission [7]. The stratification of inpatients based on the risk of re-admission can offer care providers valuable insight to modify interventions, such as discharge planning and the opportunity to influence outcomes by proactively managing high-risk patients.

Hospital re-admission risk prediction models have been traditionally developed using hypothesis-driven statistical methods since the 1980s; and as of 2015 at least 94 unique models have been described in the published literature [8,9]. Although these risk prediction models are helpful decision-making tools, their utility is limited by considerations of generalizability, adaptability, and absolute predictive performance. First, most of these models have been developed using high-quality data from selected patient cohorts and therefore can have inconsistent external validity in other settings and patient populations, in the setting of missing data and over time [10]. Second, these models require health care personnel to calculate the risk score for every patient, thereby creating barriers to their adoption. Finally, these models often cannot be adapted to incorporate information that might be of predictive value in different patient populations, resulting in the suboptimal predictive performance. In contrast, machine learning analytical methods can be used to build flexible, customized, and automated predictive models using the information available in EMRs [11]. The promise of extracting predictive insights in real time from complex and voluminous EMR data has fueled a lot of excitement around the application of machine learning–based predictive methods in health care, where even a marginal increase in the performance could translate to meaningful gains in efficiency and quality.

Predictive models developed from EMR data using machine learning methods have their own share of generalizability challenges. First, models that are developed using a large number of predictors relative to the number of outcome events are prone to overfitting. A well-known example of this is Google Flu Trends, which predicted twice the actual number of influenza-related doctor visits in 2013 [12]. Second, models developed using EMR data are subject to bias resulting from patient self-selection, confounding by indication and inconsistent availability of outcome data [13]. Finally, the practice of medicine itself evolves, thereby impacting the accuracy of predictions over time. A study determined that the relevance of clinical data used to predict future inpatient orders “decayed” with an effective half-life of about 4 months [14]. Given these limitations, it is necessary to validate the predictive performance of machine learning–based models in an independent dataset before it can be adopted in the clinical practice.

A machine learning–based model to predict 30-day re-admissions in patients with heart failure was developed at Partners HealthCare System (PHS; Boston, MA, USA) in collaboration with Hitachi, Ltd (Tokyo, Japan). Details about the development of the prediction model are described in a separate paper [15]. Briefly, the model was trained using deidentified longitudinal medical record data of 11,510 patients with heart failure who were discharged alive after an inpatient admission in the financial years 2014-2015 from the PHS. There were 27,334 inpatient admissions and 6369 30-day re-admissions during this period. The final model included 3512 variables comprising demographics, encounter, diagnosis, procedure, medication, and laboratory information as well as selected extracts from ambulatory visit notes and discharge summaries. Deep unified networks—a new mesh-like network structure of deep learning with vertical and horizontal connections of neurons to avoid overfitting—was used to develop the risk prediction model. Ten-fold cross-validation was used to validate the model internally. The model showed moderate discriminative ability with a concordance statistic of 0.71. This paper describes the protocol for independent, prospective validation of this machine learning–based model trained to predict the risk of 30-day re-admission in patients with heart failure. Hence, this study aims to prospectively validate a machine learning–based predictive model for inpatient admissions in patients with heart failure by comparing its predictions of risk for 30-day re-admissions against outcomes observed prospectively in an independent patient cohort.

## Methods

We have followed the guidelines suggested by Luo et al for reporting this protocol [16].

### Study Design

The validation of the predictive model will be conducted as a prospective cohort study. The study has been approved by the Partners Human Research Committee, the Institutional Review Board for PHS.

### Setting

The study will be conducted in 5 major hospitals that are a part of the PHS, a major health care provider in Massachusetts, USA.

### Definition of Key Variables

#### Index Admission

Every inpatient admission for a patient diagnosed with heart failure that meets the eligibility criteria as outlined in Textbox 1 will be regarded as an index admission.

#### 30-Day Re-Admission

Any inpatient admission that occurs within 30 calendar days from the date of discharge from an index admission, due to any cause, will be regarded as a 30-day re-admission. Every 30-day re-admission encounter is also regarded as a new index admission if it satisfies the eligibility criteria outlined in Textbox 1.

#### Prediction Goal

The model was trained to prognosticate the probabilities of 30-day re-admissions for every live discharge following hospital admission, based on the information available in the EMR up to the time of discharge.

**Eligibility criteria for patients and index admissions.**

- **Specific to the patient**
  1. Age 18 years or older
  2. Was not part of the dataset used to develop the algorithm
  3. Diagnosed with heart failure, with any of the following heart failure International Classification of Diseases codes assigned as a principal diagnosis code:
  - *International Classification of Diseases, Ninth Revision, Clinical Modification*
    - 402.01 Malignant hypertensive heart disease with heart failure
    - 402.11 Benign hypertensive heart disease with heart failure
    - 402.91 Unspecified hypertensive heart disease with heart failure
    - 404.01 Hypertensive heart and chronic kidney disease, malignant, with heart failure and with chronic kidney disease stage I through stage IV, or unspecified
    - 404.03 Hypertensive heart and chronic kidney disease, malignant, with heart failure and with chronic kidney disease stage V or end-stage renal disease
    - 404.11 Hypertensive heart and chronic kidney disease, benign, with heart failure and with chronic kidney disease stage I through stage IV, or unspecified
    - 404.13 Hypertensive heart and chronic kidney disease, benign, with heart failure and chronic kidney disease stage V or end-stage renal disease
    - 404.91 Hypertensive heart and chronic kidney disease, unspecified, with heart failure and with chronic kidney disease stage I through stage IV, or unspecified
    - 428 All heart failure: left; systolic, diastolic, combined; acute, chronic, acute on chronic; unspecified
  - *International Classification of Diseases, Tenth Revision, Clinical Modification*
    - 150 All heart failure: left; systolic, diastolic, combined; acute, chronic, acute on chronic; unspecified
- **Specific to the index admission**
  1. The patient was discharged alive
  2. The patient was not discharged against medical advice

Patient associated with this admission does not transfer out of the Partners HealthCare System within 30 days of discharge.

### Study Procedures

#### Identification of Eligible Index Admissions and 30-Day Re-Admissions

Customized reports will be generated daily from the EMR to alert the study staff about any heart failure patient discharged from an inpatient admission. Study staff will verify that all eligibility criteria are met, and flag index admissions associated with patients that were part of the development dataset for exclusion. The rationale of this criterion is to prevent “validation leakage,” that is, to prevent the model from making an accurate prediction based on prior knowledge about a high-risk patient acquired from the training dataset [17]. For a 30-day period following the first encounter, the EMR system will automatically notify the study staff every time any one of these patients has a subsequent encounter within the PHS. Based on these alerts, an inpatient admission due to any cause will be recorded as a 30-day re-admission.

#### Data Extraction, Processing, and Storage

Information pertaining to every eligible index admission will be extracted from two centralized data warehouses that gather clinical information from various PHS hospitals. The files will be extracted in batches every 15 days and renamed if necessary to match the naming format of files originally used to train the algorithm. All files will be stored in a HIPPA (Health Insurance Portability and Accountability Act of 1996)–compliant manner at the study site.

#### Obtaining Predictions From the Model

The algorithm will be housed in a server dedicated for this project at the study site. After the initial set-up is complete, the algorithm will use all files pertaining to an eligible index admission as input and process them automatically to generate variables needed for the predictive process to run. It will then provide the output in the form of a text file with a probability value assigned for every index admission.

### Statistical Analysis

#### Sample Size Calculation

Collins et al recommend a minimum of 100 events for externally validating a prognostic model [17]. The 30-day re-admission rate for heart failure index admissions in the PHS in financial years 2015-16 was 20.4%. Therefore, a minimum of 100/20.4%=491 index admissions will be required to conduct a validation study. We assume that up to 25% of re-admissions may occur outside PHS. Of these, we assume that about 50% of the admissions will be from patients who were part of the dataset used for the development of the algorithm and therefore will have to be excluded. Therefore, a total sample size of 1228 index admissions may have to accrue before we observe 100 eligible events (ie, 30-day re-admissions).

#### Evaluation of Model Performance

##### Evaluation of Discrimination Thresholds

The output provided by the model is a probability score for 30-day re-admission for each index admission. Multiple probability scores will be evaluated to determine the threshold that is the most optimal binary classifier of index admissions at-risk or not-at-risk for 30-day re-admissions. The following metrics will be used to evaluate thresholds:

1. Sensitivity: True positives/(true positives + false negatives)
2. Specificity: True negatives/(true negatives + false positives)
3. Positive predictive value (PPV): True positives/(true positives + false positives)
4. Negative predictive value (NPV): True negatives/(true negatives + false negatives)
5. Accuracy: Number of correct assessments (true positives + true negatives)/number of assessments

##### Evaluation of Overall Performance of the Model

The overall performance of the model was evaluated by:

1. Concordance statistic (C-index): This is equal to the area under the receiver operating characteristic curve, which is a plot of the true positive rate (sensitivity) against the false positive rate (1-specificity) at various discrimination threshold settings.
2. Model calibration: This refers to the agreement between the predictions made by the model and the observed outcomes. We will use contingency tables and survival plots to assess the relationship between predicted risk and observed 30-day re-admission rates.
3. Brier score: 1/N Σ(f_t_−o_t_)^2^, where *N* is the number of index cases, *f*_t_ is the forecast probability, and *o*_t_ is the outcome (1 if it happened, 0 if it did not). The Brier score measures the accuracy of a forecast [18]. The best possible score is 0.

## Results

The project was funded in April 2017 and data collection began in June 2017. Enrollment was completed in July 2017. Data analysis is currently underway, and the first results are expected to be submitted for publication in October 2018.

## Discussion

### Principal Findings

Our study will prospectively evaluate the performance of a machine learning model that predicts the risk of 30-day re-admissions for patients with heart failure in a real-world hospital system. Risk prediction models are designed to aid clinical decision making, and in the context of heart failure, their implementation can potentially result in substantial reductions in rehospitalizations and cost savings [19]. Risk prediction models are not new to medicine, and there is no dearth of models developed using traditional statistical techniques [8,20]. However, the clinical adoption of risk prediction models remains quite low [21,22]. Some of the barriers reported by physicians for not using risk prediction models are lack of time, lack of trust in its validity, and uncertainty about generalizability to the specific patient population observed by an individual physician [21,22]. Machine learning models are well-placed to overcome these barriers. Automation is a fundamental feature of machine learning–based prediction models, thereby eliminating the need for input from the provider to calculate risk scores for every patient. Moreover, machine learning models can be “fine-tuned” for different patient populations and even individual hospital systems, such that the prediction results are most generalizable to that population. Building a one size fits all prediction model that is generalizable to every hospital system is neither a desirable goal for a metric such as 30-day re-admissions, which reflects the quality of care at a particular hospital, nor an efficient utilization of the ability of machine learning analytical techniques to extract fine-grained information from thousands of variables, the “richness” of which may vary from one institution to another.

In this study, we will include any re-admission as an index admission in the analysis, as long as it meets the inclusion criteria for index admissions; this is similar to the method used for training the predictive model [15], and aligns with the definition used by the Centers for Medicare and Medicaid Services for 30-day all-cause re-admission rates [23]. This choice was made keeping in mind the intended real-world use of the model where it will be applied to every inpatient admission. The consequent relative increase in the prevalence of the outcome (ie, 30-day re-admissions) can be expected to result in higher sensitivity and PPV and lower specificity and NPV compared with a definition that does not allow re-admissions to be considered as index admission. We expect the impact on PPV and NPV to be substantially higher than that on the sensitivity and specificity because the former are prevalence-dependent metrics. We do not expect any change in the area under the receiver operating characteristic curve, calibration, and Brier score.

Rigorous evaluation of the validity of machine learning models is an important step to address barriers to the clinical adoption of these models; this information is valuable not only to better inform physicians but also to help hospital administrators in estimating the cost-effectiveness of investing into a machine learning–based prediction system. The 30-day re-admission rates vary across hospitals based on the sociodemographic profile of patients, access to care, and the case-mix of patients, among other factors [24]. Thus, predictive models that are generalizable across health care systems might result in the suboptimal utilization of information that might be of predictive value within a given hospital system. To ensure that predictive models “fine-tuned” to specific health care systems are dependable, prospective validation studies conducted periodically in independent patient samples should become the norm in the evaluation of machine learning–based prediction algorithms. The results from such studies will help detect the true performance of the model and estimate the frequency at which the algorithm needs to be fine-tuned.

### Limitations and Strengths

This study has certain limitations. The use of this prediction model in the real world is, by design, intended to effect a change in the behavior of providers. As the prediction results from our model will not be available to physicians, this prospective validation cannot estimate any changes in the model’s performance under such circumstances. In addition, we cannot detect re-admissions that may occur outside of PHS; this may result in an underestimation of the model performance. However, the model was trained using the same constraint. Thus, we do not expect attenuation in the model performance because of this constraint, compared with its performance after training.

This study also has several important strengths. To the best of our knowledge, this is the first study to prospectively evaluate a machine learning predictive model in a real-world hospital setting, and we hope that the detailed procedures described here will enable the design of similar studies to evaluate the performance of other machine learning predictive models. Second, in addition to the C-statistic, we also evaluate the sensitivity, specificity, PPV, and NPV of the machine learning predictive model. These metrics take the prevalence of the outcome into account, unlike the C-statistic that is independent of prevalence [25], and therefore have greater clinical relevance. Past studies have showed that PPV and C-statistic have minimal correlation for risk prediction models [26]. Finally, we evaluate the performance of the model 2 years after it was built and exclude patients who were part of the training dataset. These steps are essential to eliminate validation leakage and help to estimate the stability of the model over time.

### Conclusions

The application of machine learning–based algorithms to diagnose diseases, prognosticate outcomes, and personalize treatments is increasing. Rigorous evaluation of their performance is critical for the widespread adoption. Findings from this study may better inform decisions related to the application of machine learning solutions in health care.

## Abbreviations

AI: artificial intelligence
EMR: electronic medical record
HIPPA: Health Insurance Portability and Accountability Act
NPV: negative predictive value
PHS: Partners HealthCare System
PPV: positive predictive value
